# Insulin Resistance and Obesity Affect Lipid Profile in the Salivary Glands

**DOI:** 10.1155/2016/8163474

**Published:** 2016-07-13

**Authors:** Jan Matczuk, Anna Zalewska, Bartłomiej Łukaszuk, Małgorzata Knaś, Mateusz Maciejczyk, Marta Garbowska, Dominika M. Ziembicka, Danuta Waszkiel, Adrian Chabowski, Małgorzata Żendzian-Piotrowska, Krzysztof Kurek

**Affiliations:** ^1^County Veterinary Inspection, 26B Zwycięstwa Street, 15-959 Białystok, Poland; ^2^Department of Conservative Dentistry, Medical University of Bialystok, 1 Kilińskiego Street, 15-222 Białystok, Poland; ^3^Department of Physiology, Medical University of Bialystok, 2C Mickiewicza Street, 15-222 Białystok, Poland; ^4^Institute of Health Care Higher Vocational School, 10 Noniewicza Street, 16-400 Suwałki, Poland; ^5^Students' Scientific Group “Stomatological Biochemistry”, Department of Conservative Dentistry, Medical University of Bialystok, 1 Kilińskiego Street, 15-222 Białystok, Poland; ^6^Department of Hygiene, Epidemiology and Ergonomics, Medical University of Bialystok, 2C Mickiewicza Street, 15-222 Białystok, Poland; ^7^Department of Public Health, Medical University of Bialystok, 1 Kilińskiego Street, 15-222 Białystok, Poland

## Abstract

In today's world wrong nutritional habits together with a low level of physical activity have given rise to the development of obesity and its comorbidity, insulin resistance. More specifically, many researches indicate that lipids are vitally involved in the onset of a peripheral tissue (e.g., skeletal muscle, heart, and liver) insulin resistance. Moreover, it seems that diabetes can also induce changes in respect of lipid composition of both the salivary glands and saliva. However, judging by the number of research articles, the salivary glands lipid profile still has not been sufficiently explored. In the current study we aim to assess the changes in the main lipid fractions, namely, triacylglycerols, phospholipids, free fatty acids, and diacylglycerols, in the parotid and the submandibular salivary glands of rats exposed to a 5-week high fat diet regimen. We observed that the high caloric fat diet caused a significant change in the salivary glands lipid composition, especially with respect to PH and TG, but not DAG or FFAs, classes. The observed reduction in PH concentration is an interesting phenomenon frequently signifying the atrophy and malfunctions in the saliva secreting organs. On the other hand, the increased accumulation of TG in the glands may be an important clinical manifestation of metabolic syndrome and type 2 diabetes mellitus.

## 1. Introduction

Carbohydrates and lipids are the two most important classes of molecules in respect of the body energy provisions. In today's world, however, wrong nutritional habits together with a low level of physical activity have given rise to the development of obesity and its comorbidity, insulin resistance (IR). More specifically, a lot of research indicates that lipids are vitally involved in the onset of a peripheral tissue (e.g., skeletal muscle, heart, liver, and adipose tissue) insulin resistance [1–3]. The latter seems to occur in relation to an imbalance between superfluous cellular free fatty acids (FFAs) supply and their inadequate utilization in the process of mitochondrial *β*-oxidation [4]. Moreover, many of the previously conducted studies have confirmed the perturbing effects of FAs or their cellular intermediates, on the insulin signaling pathway [5], whereas palmitic acid is commonly applied to evoke IR in both animals [6] and cell lines [7]. Furthermore, the existence of a positive correlation between intramyocellular lipids content and the degree of insulin resistance has been strongly established in the scientific literature. In addition, triacylglycerol (TG) and diacylglycerol (DAG) are probably the two most commonly discussed, with respect to lipid-induced deterioration of insulin sensitivity, lipids fractions [4, 8]. Interestingly, some research has indicated that the accumulation of a various size cytoplasmic lipid droplets occurs also in the diabetic rat's salivary glands [9, 10].

The main function of the salivary glands is, unsurprisingly, the synthesis of saliva, a watery fluid containing electrolytes, mucus, and enzymes. Alongside its role in digestion saliva serves also some protective functions in respect of the oral mucosa and gingiva. Moreover, research of Tomita et al. indicates that the secretions of the parotid and the submandibular salivary glands contain also lipid component (in an amount of about 5–10 mg per 100 mL of the secretion) [11]. Interestingly, not only do the salivary glands play an important role in respect of oral hygiene maintenance, but also they are associated with hyperglycemia and other metabolic disturbances occurring after the onset of diabetes [12, 13]. Moreover, Sandberg et al. showed that oral health problems are the health complications affecting approximately half the people with hyperglycemia [14]. Other common diabetes codiseases/coailments include periodontitis, gingivitis, xerostomia (dry mouth syndrome), tooth decay (also known as dental caries) and loss, and lesions of the tongue and the oral mucosa [15, 16]. Furthermore, diabetes also seems to be associated with both the parotid and the submandibular salivary glands atrophy, as confirmed by the glands weight and size reductions accompanied by a degeneration of the acinar cells and a decrement in the secretory granules diameter [17–19].

Previously conducted research indicates that diabetes frequently leads to the imbalance in the salivary glands lipid metabolism which subsequently results in the cytoplasm lipid droplets accumulation. However, judging by the limited number of the research articles, the salivary glands lipid profile still has not been sufficiently explored. In the current study we aim to assess, in detail, changes in the main lipid fractions, namely, triacylglycerols, phospholipids, free fatty acids, and diacylglycerols, concentration in the parotid (PSG) and the submandibular (SMSG) salivary glands of rats exposed to a 5-week high fat diet regimen.

## 2. Materials and Methods

### 2.1. Experimental Model

The research was conducted on male Wistar rats randomly assigned to one of the experimental groups (8 specimens in each group). Prior to any experiments all procedures concerning animal treatment and maintenance were approved by the Local Ethical Committee for Animal Experiments of the Medical University of Bialystok. The rats were maintained in the appropriate conditions. Throughout the experiment a stable temperature (21-22°C), humidity, twenty-four-hour rhythm (12 h/12 h light-dark cycle), and free access to food and water were preserved.

At the beginning of the experiment the animals were randomly allocated into one of the two groups: control (C): with unrestricted access to a standard rodent diet; and high fat diet fed group (HFD): with unrestricted access to a high caloric research diet (60% of energy derived from fats, as described before [3]). All tissue collection procedures were performed at the beginning of the 6th week; prior to it the animals were fasted overnight and subsequently intraperitoneally injected with pentobarbital (80 mg/kg of body weight). The parotid and the submandibular salivary glands samples were cut out, afterwards immediately frozen, and stored in liquid nitrogen for the time of further analyses.

### 2.2. Blood Parameters

 In addition to the abovementioned procedures also blood from the abdominal aorta was collected. The blood was destined for the analysis of the fasting glucose (Accu-check glucometer, Byer, Germany), insulin (chemiluminescence, Abbot, USA), and free fatty acids levels (as described by Bligh and Dyer [20]). Based on these assessments we have estimated the animals' insulin resistance using widely accepted HOMA-IR formula; that is, HOMA = glucose *∗* insulin/405.

### 2.3. Salivary Glands Lipids Composition

According to the protocol [20], four previously selected lipid fractions, that is, triacylglycerols, phospholipids, free fatty acids, and diacylglycerols, concentrations were assessed. For this reason the excised salivary glands samples were pulverized by grinding them in a liquid nitrogen precooled mortar and pestle. The obtained tissue powder was then moved to glass tubes for the subsequent lipids extraction according to the protocol developed by Bligh and Dyer [20]. The TG, DAG, and PH fractions separations were performed on the basis of thin-layer chromatography [3]. For the identification and quantification of individual fatty acid methyl esters measurements of the gas-liquid chromatography standards retention times were applied. The abovementioned were run on a Hewlett-Packard 5890 Series II gas chromatograph, with a Varian CP-SIL capillary column (50 m × 0.25 mm internal diameter) and flame-ionization detector (FID) (Agilent Technologies, USA). In the abovementioned measurements the following standards were used: (C 17:0) heptadecanoic acid, as a standard for FFAs quantification; (C 17:0) 1,2-diheptadecanoin, as a standard for DAG quantification; (C 17:0) triheptadecanoin, as a standard for TG quantification; (C 17:0) 1,2-diheptadecanoyl-sn-glycero-3-phosphocholine, as a standard for PH quantification. Total lipid content in each class (TG, PH, FFAs, and DAG) is expressed in nanomoles per gram of the tissue and was calculated from the sum of the particular fatty acid species in a given class.

### 2.4. Statistical Analysis

Between groups differences were detected using unpaired Student's *t*-test (*α* set at 0.05). In the case of data distribution other than normal and/or variance heteroscedasticity the *U* test (Mann-Whitney) was applied. All of the obtained results are presented in tables and in the form of bar-plots with the mean as a bar height and whiskers as the standard deviation (1 SD) value. Sample size was set based on a previously conducted pilot study; the power of the test was set at 0.8.

## 3. Results

### 3.1. Effects of High Fat Diet Feeding on the Body Weight, Glucose Homeostasis, and Plasma Free Fatty Acids Level (Table 1)

The average daily food intake was similar in both studied groups. The high fat diet fed rats were characterized by a significantly increased body mass, as compared with the control group (*p* < 0.05). Moreover, the high fat diet feeding also affected glucose homeostasis. We noticed an increase in the fasting glucose level as well as the level of insulin in the HFD group in comparison with the control animals (*p* < 0.05 and *p* < 0.05, resp.). Furthermore, we observed that the high fat diet feeding led to the development of insulin resistance, as assessed based on the elevated HOMA-IR index, in the HFD group in comparison with the control group (*p* < 0.05). Finally, in comparison with the control group the rats fed with the high fat diet were characterized by an increased plasma free fatty acids concentration (*p* < 0.05).

### 3.2. Effects of High Fat Diet Feeding on PH Content in the Salivary Glands (Figure 1, Tables 2 and 3)

In the parotid salivary glands we did not observe any significant differences in total PH concentrations between the C and HFD group. However, we noticed an increment of oleic (18:1), linoleic (18:2), eicosapentaenoic (20:5), and docosahexaenoic (22:6) acids concentration in the HFD group in comparison with the C group (*p* < 0.05).

On the contrary, the submandibular salivary glands total PH content was significantly decreased (*p* < 0.05) in the HFD group compared with the C group. Furthermore, we found that in the HFD group in comparison with the C group both saturated and unsaturated fatty acids contents were decreased (*p* < 0.05). Among the unsaturated fatty acids in the HFD group compared with the C group the following fractions were decreased: palmitoleic (16:1), oleic (18:1), linoleic (18:2), *α*-linoleic (18:3), arachidonic (20:4), nervonic (24:1), eicosapentaenoic (20:5), and docosahexaenoic (22:6) (*p* < 0.05). Moreover, among the saturated fatty acids myristic (14:0), palmitic (16:0), stearic (18:0), arachidic (20:0), and behenic (22:0) acid were significantly decreased in the HFD group in comparison with the C group (*p* < 0.05).

### 3.3. Effects of High Fat Diet Feeding on FFAs Content in the Salivary Glands (Figure 1, Tables 4 and 5)

In the parotid salivary glands total FFAs content was significantly decreased (*p* < 0.05) in the HFD group, as compared with the C group. Moreover, we found that in the HFD group in comparison with the C group both saturated and unsaturated fatty acids contents were decreased (*p* < 0.05). Among the unsaturated fatty acids in the HFD group compared with the C group the following fatty acid species were decreased: palmitoleic (16:1), oleic (18:1), linoleic (18:2), *α*-linoleic (18:3), arachidonic (20:4), eicosapentaenoic (20:5), nervonic (24:1), and docosahexaenoic (22:6) (*p* < 0.05). Moreover, among the saturated fatty acids myristic (14:0), palmitic (16:0), stearic (18:0), behenic (22:0), and lignoceric (24:0) acids were significantly decreased in the HFD group in comparison with the C group (*p* < 0.05).

Also in the submandibular salivary glands total FFAs content was significantly decreased (*p* < 0.05) in the HFD group, as compared with the C group. Furthermore, we found that in the HFD group in comparison with the C group both saturated and unsaturated fatty acids contents were decreased (*p* < 0.05). Among the unsaturated fatty acids in the HFD group compared with the C group the following fatty acid species were decreased: palmitoleic (16:1), oleic (18:1), linoleic (18:2), *α*-linoleic (18:3), arachidonic (20:4), eicosapentaenoic (20:5), nervonic (24:1), and docosahexaenoic (22:6) (*p* < 0.05). Moreover, among the saturated fatty acids myristic (14:0), palmitic (16:0), stearic (18:0), arachidic (20:0), behenic (22:0), and arachidonic acid (24:0) were significantly decreased in the HFD group in comparison with the C group (*p* < 0.05).

### 3.4. Effects of High Fat Diet Feeding on DAG Content in the Salivary Glands (Figure 1, Tables 6 and 7)

In the parotid salivary glands total DAG content was significantly decreased (*p* < 0.05) in the HFD group, as compared with the C group. Moreover, we found that in the HFD group in comparison with the C group both saturated and unsaturated fatty acids contents were decreased (*p* < 0.05). Among the unsaturated fatty acids in the HFD group compared with the C group the following fatty acid species were decreased: palmitoleic (16:1), oleic (18:1), linoleic (18:2), *α*-linoleic (18:3), arachidonic (20:4), and docosahexaenoic acid (22:6) (*p* < 0.05). Moreover, among the saturated fatty acids myristic (14:0), palmitic (16:0), stearic (18:0), and behenic acid (22:0) were significantly decreased in the HFD group in comparison with the C group (*p* < 0.05).

Also in the submandibular salivary glands total DAG content was significantly decreased (*p* < 0.05) in the HFD group, as compared with the C group. Furthermore, we found that in the HFD group in comparison with the C group both saturated and unsaturated fatty acids contents were decreased (*p* < 0.05). Among the unsaturated fatty acids in the HFD group compared with the C group the following fatty acid species were decreased: palmitoleic (16:1), oleic (18:1), linoleic (18:2), *α*-linoleic (18:3), arachidonic (20:4), eicosapentaenoic (20:5), nervonic (24:1), and docosahexaenoic acid (22:6) (*p* < 0.05). Moreover, among the saturated fatty acids myristic (14:0), palmitic (16:0), stearic (18:0), arachidic (20:0), and behenic acid (22:0) were significantly decreased in the HFD group in comparison with the C group (*p* < 0.05).

### 3.5. Effects of High Fat Diet Feeding on TG Content in the Salivary Glands (Figure 1, Tables 8 and 9)

In the parotid salivary glands we did not observe any significant differences in total TG concentrations between the C and HFD groups. However, we noticed an increment of linoleic (18:2), eicosapentaenoic (20:5), and docosahexaenoic acid (22:6) concentration coexisting with a decrement of behenic acid content (22:0) in the HFD group in comparison with the C group (*p* < 0.05).

On the contrary, the submandibular salivary glands total TG content was significantly increased (*p* < 0.05) in the HFD group compared with the C group. Among the unsaturated fatty acids in the HFD group compared with the C group the fatty acid species, oleic (18:1), eicosapentaenoic (20:5), and docosahexaenoic acid (22:6), contents were increased (*p* < 0.05), whereas palmitoleic (16:1) and nervonic acid (24:1) contents were decreased (*p* < 0.05). Moreover, among the saturated fatty acids myristic (14:0) and arachidonic acid (24:0) contents were significantly increased, whereas the quantity of stearic acid (18:0) was decreased in the HFD group in comparison with the C group (*p* < 0.05).

## 4. Discussion

Obesity is currently a predominant medical condition because for several dozens of years an alarming increase in its prevalence has been observed. Unfortunately, raised body mass index (BMI) is an important risk factor for the development of several diseases, including insulin resistance and type 2 diabetes. Tissues which are responsible for the onset and development of the aforementioned conditions are mainly skeletal muscles, liver, and white adipose tissue. However, glucose homeostasis imbalance may affect any other tissue, including the salivary glands. Therefore, in the present study we examined the effect(s) of a diet induced insulin resistance and obesity on lipid profile in both the parotid and the submandibular salivary glands.

Some of the previously published reports indicate that chronic high fat diet feeding causes hyperglycemia which can contribute to the accumulation of lipid droplets in the cytoplasm of many nonadipose tissues, including skeletal muscles, liver, and even salivary glands [3, 9, 10]. Therefore, not surprisingly, our 5-week high fat diet feeding has led to a significantly greater animals' body mass which was accompanied by increased levels of blood glucose, insulin, and plasma FFAs together with the build-up in the salivary glands lipid concentration. All of the abovementioned changes were in accordance with previously conducted research [3]. Moreover, based on the HOMA-IR values we can claim that the prolonged high fat diet feeding decreased the whole body insulin sensitivity. Finally, although not tested here, it is widely acknowledged that FFAs exert a negative influence in respect of the regulation of cellar glucose uptake and glycogen synthesis, thus leading to the development of hyperglycemia (as observed in our HFD group) [2]. It is important to emphasize that chronic high fat diet feeding may cause a reduction in unstimulated and stimulated salivary flow rate. In the group of obese patients disturbances in the oxidant/antioxidant homeostasis were previously observed [21]. On the other hand, the parotid and the submandibular glands of rats react differently to insulin resistance conditions, with more pronounced changes observed in the submandibular glands [22]. The observed different responsiveness of the glands to high fat diet feeding is not easy to explain; it was, however, observed in some other studies [23]. Ibuki et al. [23], for instance, showed that there was a significant decline in the submandibular salivary glands mass in the time-course of diabetes. Moreover, the authors reported an increased level of MCD (malondialdehyde which is a marker of increased lipid peroxidation) with the changes more pronounced in the case of the SMSG. The observed different responsiveness could be, perhaps, a reflection of a different tissue metabolism (PSG, oxidative, SMSG, glycolytic) [23]. If so, then the response pattern of the salivary glands (to high fat diet feeding) differs from the one observed in skeletal muscles where more pronounced changes in lipid metabolism are observed in the case of the more oxidative ones (e.g., soleus versus white gastrocnemius) [3].

Scientific investigation has led to the discovery of more than 30 different biological components present in the salivary glands, of which lipids seem to be a particularly important representative [24, 25]. Phospholipids, for instance, are the most abundant component in a plasmalemma and are vitally involved in the transport processes taking place across this biological membrane. Interestingly, according to some previous research several pathological conditions, that is, diabetes, aging, or weaning, may contribute to the decreased salivary glands PH content. Tomita et al., for example, indicated a probable negative correlation between the salivary glands PH concentration and the age of animals [11]. This phenomenon could be of particular importance to the discussed topic given commonly observed age-progressing decrement of peripheral tissues insulin sensitivity and the reduction of PH content observed in this study. Moreover, also data from Kamata et al. seem to indirectly confirm this assumption [26]. In the aforementioned investigation the authors reported that type 1 diabetes resulted in a degeneration of the salivary glands acinar cells together with a concomitant decline in their secretory granules number. The researchers postulated that the observed results could be, at least partially, evoked by a significant reduction in PH concentration, which in turn could negatively impact cell membranes stability [26]. Therefore, it seems conceivable that the changes in the salivary glands PH content, as observed by us and other authors, might lead to the detrimental changes in the salivary glands structure, which in turn could be reflected in their functions, for example, by causing a decreased production of saliva, that is, hyposalivation.

On opposition to the above-discussed phospholipid fraction free fatty acids are believed to be heavily implicated in the processes of intracellular signal transmission. Previously published observations performed on insulin resistant diabetic animals clearly demonstrated increased FFAs concentrations in many tissues, including skeletal muscles and liver [2, 3]. Increased intracellular FFAs content strongly affects insulin signaling pathway and contributes to insulin resistance condition. Surprisingly, in the present study we observed, despite the increased blood FFAs supply, that the insulin resistant diabetic animals were characterized by a decreased FFAs content in both the parotid and the submandibular salivary glands. Furthermore, we found that the feeding with high fat diet led to a decrement in both saturated and unsaturated fatty acids contents. This phenomenon could be explained by either a decreased FFAs uptake from the blood or their transformation into other lipid fractions (perhaps TG, since their concentration increased significantly in the submandibular glands of the rats fed with high fat diet). Furthermore, it is well established that the fatty acid synthesis is a multistep process, with one of the steps, desaturation, catalyzed by desaturases. Interestingly, it has been shown that the activity of desaturases, including those originating from the salivary glands, decreases in the absence of insulin or during insulin resistance. As an end result of the decreased desaturase activity a decline in the number and amount of some unsaturated FFAs species occurs, since desaturase introduces “unsaturated” double carbon bond into a fatty acid chain. Similar findings were also reported by Mahay et al. [27]; however, not all research groups confirm them [28].

Interestingly, in the present study we have noticed a decreased level of DAG in the parotid and the submandibular salivary glands of the diabetic rats. We demonstrated, likewise in the case of FFAs, that this decrement applies to both the saturated and the unsaturated fatty acid species. At first glance, this novel and intriguing finding seems to be quite unexpected. Many authors point on the causative relationship between DAG over accumulation and insulin resistance in peripheral tissues (e.g., skeletal muscles, adipose tissue, and liver) [2, 4]. However, we should be aware that the salivary glands are not as vitally involved in the development of this metabolic condition as the aforementioned tissues (due to their relatively large mass and high metabolic activity) are. Moreover, closer literature data review indicates that in the salivary glands DAG intracellular signaling may contribute to the muscarinic-induced saliva secretion; thus any reduction in its (DAG) concentration would result in a hyposalivation, a condition observed in both human and animal diabetic individuals [29].

**Figure 1:**
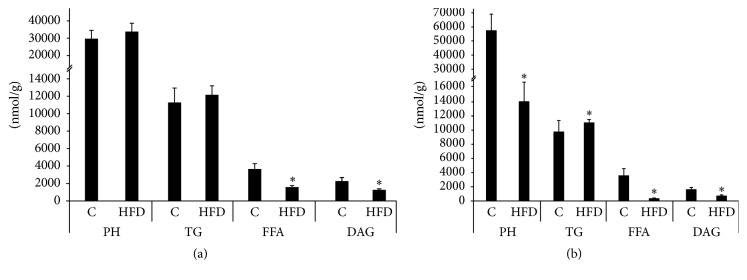
Effects of high fat diet feeding on the salivary glands lipid profile ((a) parotid salivary glands; (b) submandibular salivary glands). Ctrl: control group (*n* = 8); DG: diacylglycerols; FFAs: free fatty acids; HFD: high fat diet fed group (*n* = 8); PH: phospholipids; PSG: parotid salivary glands; SMSG: submandibular salivary glands; TG: triacylglycerols; *∗*: difference versus Ctrl (*p* < 0.05).

Another plausible explanation for the observed reductions in the salivary glands lipid content is a possible tissue atrophy frequently observed in some of the previous studies [19, 23]. Although the aforementioned studies tested the glands metabolism in the case of rats with streptozotocin induced diabetes they do, however, share significant similarities with the model applied by us. With respect to the discussed topic it is important to note that both type 1 and type 2 diabetes are characterized by elevated levels of serum glucose and free fatty acids concentrations [3, 30]; thus the cellular external environment is somewhat alike. Ibuki et al. [23], for instance, showed that there was a significant decline in the submandibular, but not the parotid, salivary glands mass between the 28th and 45th day of the experiment (our experiment (HFD) lasted 5 weeks, that is, 35 days). Moreover, the authors reported increased level of MCD (malondialdehyde, which is a marker of increased lipid peroxidation) in both of the examined glands. This could indicate an increased cellular atrophy in the tissues and therefore increased lipids degeneration. The possibility of such cellular atrophy was not directly tested in our study; however, if it were the case one would expect it to be visible in all of the examined lipid fractions. According to our data (Tables 2 [Table tab3] [Table tab4] [Table tab5] [Table tab6] [Table tab7] [Table tab8]–9, Figure 1), though, it seems that it did not happen in the case of the two most abundant lipid classes: PH (a decrease only in the submandibular salivary glands) and TG fractions (no difference in the case of PSG and an increase in the case of SMSG). Therefore, it is possible that the observed changes are not the result of the cellular atrophy itself but may reflect the diabetic condition in a broader context.

Some of the previously published studies indicate that the salivary glands intraorgan lipid accumulation occurs along with the time-course of insulin resistance and diabetes; moreover, its magnitude usually accompanies the changes in the serum glucose concentration [28, 31]. Furthermore, immunohistochemical scrutiny revealed that these lipid droplets are composed mostly of triacylglycerol that had been, probably, accumulated as a consequence of its increased generation and/or decreased utilization for secretory purposes [31]. It has been proven that the intracellular TG accumulation is associated with insulin resistance, although nowadays this phenomenon is recognized more as a marker of accumulated lipids rather than a direct cause of type 2 diabetes. Surprisingly, Morris and coworkers discovered that even two weeks of streptozotocin induced diabetes may lead to the pronounced salivary glands lipids accumulation as confirmed by histological evaluation [28]. Interestingly, the aforementioned changes were easily reversible after barely 1 week of insulin administration. Thus, it appears that the salivary glands lipid accumulation occurs quite early in the development of diabetes and, at least at that time point, is fairly easily reversible [28]. In the present study we have demonstrated that an increment in the salivary glands TG accumulation (greater TG content in the submandibular salivary glands) may also occur in the time-course of insulin resistance and type 2 diabetes. Therefore, it has been postulated that the pronounced build-up in the salivary glands lipid content (mostly in TG fraction) not only is associated with high BMI, fatty liver disease, or coronary artery disease risk factors, but also may be a relevant clinical symptom of metabolic syndrome and/or type 2 diabetes [32].

In conclusion, due to the scarcity of the literature data the precise effects of insulin resistance and type 2 diabetes with respect to the salivary glands lipid profile were not, so far, extensively elucidated. In the present study we aimed to fill this gap. Firstly, we observed that the high fat diet regimen had caused significant changes in the salivary glands lipid composition, especially in regard to PH and TG, but not DAG or FFAs, classes. The observed reduction in PH concentration is an interesting phenomenon frequently signifying the atrophy and malfunctions in the saliva secreting organs. On the other hand, the increased accumulation of TG in the glands may be an important clinical manifestation of metabolic syndrome and type 2 diabetes mellitus.

## Figures and Tables

**Table 1:** Effect of high fat diet feeding on body weight, fasting serum glucose level, fasting serum insulin level, the Homeostasis Model Assessment of Insulin Resistance (HOMA-IR) index, and serum FFA level (measured at the beginning of the 6th week).

	C	HFD
Body weight (g)	315.6 ± 17.0	375.4 ± 18.1^*∗*^
Glucose level (mg/dL)	101.3 ± 6.4	164.5 ± 12.4^*∗*^
Insulin level (*µ*U/mL)	4.6 ± 0.6	55.7 ± 5.7^*∗*^
HOMA-IR	1.6 ± 1.1	20.0 ± 2.5^*∗*^
FFA level (*µ*mol/L)	88.6 ± 10.4	152.4 ± 10.1^*∗*^

C: control group. HFD: group fed with high fat diet.

Results are based on 8 independent preparations for each experimental treatment (means ± SD).

^*∗*^ *p* < 0.05 compared with C group.

**Table 2:** Phospholipids composition in the parotid salivary glands (nmol/g).

Fatty acid	Control	High fat diet
Myristic (14:0)	1067.79 ± 245.404	1064.25 ± 183.734
Palmitic (16:0)	18308.42 ± 3549.057	20006.66 ± 3724.717
Palmitoleic (16:1)	2262.11 ± 752.999	1716.34 ± 255.614
Stearic (18:0)	1666.38 ± 290.83	1936.75 ± 250.423
Oleic (18:1)	3515.13 ± 567.385	4726.36 ± 910.837^*∗*^
Linoleic (18:2)	1920.61 ± 575.49	2656.63 ± 432.943^*∗*^
Arachidic (20:0)	101.46 ± 21.18	118.36 ± 36.992
*α*-linoleic (18:3)	257.25 ± 90.915	212.67 ± 30.417
Behenic (22:0)	167.36 ± 28.941	131.77 ± 32.863
Arachidonic (20:4)	737.81 ± 101.194	731.71 ± 123.755
Lignoceric (24:0)	98.77 ± 18.389	120.46 ± 37.501
Eicosapentaenoic (20:5)	30.46 ± 8.774	55.63 ± 8.098^*∗*^
Nervonic (24:1)	88.62 ± 13.577	80.72 ± 19.464
Docosahexaenoic (22:6)	451.08 ± 69.753	614.43 ± 175.756^*∗*^
UFA	9263.07 ± 971.175	10794.47 ± 1410.346
SFA	21410.18 ± 3888.879	23378.25 ± 3956.379
Total	30673.25 ± 4728.9	34172.72 ± 4828.508

Results are based on 8 independent preparations for each experimental treatment (means ± SD). ^*∗*^ *p* < 0.05 compared with control group.

**Table 3 tab3:** Phospholipids composition in the submandibular salivary glands (nmol/g).

Fatty acid	Control	High fat diet
Myristic (14:0)	1812.03 ± 410.021	220.06 ± 50.003^*∗*^
Palmitic (16:0)	20700.35 ± 5080.428	4084.86 ± 826.012^*∗*^
Palmitoleic (16:1)	4888.46 ± 1330.273	347.52 ± 90.284^*∗*^
Stearic (18:0)	2551.09 ± 568.847	1235.59 ± 228.771^*∗*^
Oleic (18:1)	15946.36 ± 3926.205	4915.29 ± 1193.102^*∗*^
Linoleic (18:2)	10439.62 ± 3124.438	2841.45 ± 698.514^*∗*^
Arachidic (20:0)	46.67 ± 14.163	31.41 ± 3.803^*∗*^
*α*-linoleic (18:3)	488.17 ± 261.042	157.67 ± 37.696^*∗*^
Behenic (22:0)	51.8 ± 7.359	32.97 ± 2.694^*∗*^
Arachidonic (20:4)	391.03 ± 70.611	196.61 ± 18.14^*∗*^
Lignoceric (24:0)	14.08 ± 3.693	12.93 ± 2.181
Eicosapentaenoic (20:5)	15.41 ± 3.307	18.88 ± 1.696^*∗*^
Nervonic (24:1)	13.09 ± 2.719	5.57 ± 0.947^*∗*^
Docosahexaenoic (22:6)	92.91 ± 26.462	38.75 ± 5.619^*∗*^
UFA	32275.05 ± 6588.666	8521.74 ± 1983.94^*∗*^
SFA	25176.03 ± 5704.184	5617.82 ± 1005.162^*∗*^
Total	57451.08 ± 11482.449	14139.56 ± 2787.592^*∗*^

Results are based on 8 independent preparations for each experimental treatment (means ± SD). ^*∗*^ *p* < 0.05 compared with control group.

**Table 4 tab4:** Free fatty acids composition in the parotid salivary glands (nmol/g).

Fatty acid	Control	High fat diet
Myristic (14:0)	73.74 ± 11.539	38.11 ± 4.69^*∗*^
Palmitic (16:0)	1225.19 ± 312.786	721.51 ± 90.679^*∗*^
Palmitoleic (16:1)	475.43 ± 90.237	87.46 ± 21.989^*∗*^
Stearic (18:0)	430.62 ± 120.932	230.14 ± 43.29^*∗*^
Oleic (18:1)	369.43 ± 50.221	156.9 ± 39.858^*∗*^
Linoleic (18:2)	337.67 ± 50.205	151.54 ± 35.83^*∗*^
Arachidic (20:0)	6.67 ± 1.861	6.25 ± 0.806
*α*-linoleic (18:3)	31.86 ± 4.276	11.21 ± 3.25^*∗*^
Behenic (22:0)	18.35 ± 4.609	4.2 ± 0.822^*∗*^
Arachidonic (20:4)	558.74 ± 242.799	136.16 ± 41.227^*∗*^
Lignoceric (24:0)	5.3 ± 1.381	3.97 ± 0.72^*∗*^
Eicosapentaenoic (20:5)	28.94 ± 17.181	9.99 ± 3.425^*∗*^
Nervonic (24:1)	3.88 ± 0.962	2.15 ± 0.29^*∗*^
Docosahexaenoic (22:6)	70.78 ± 7.997	5.88 ± 1.923^*∗*^
UFA	1876.72 ± 267.732	561.29 ± 134.462^*∗*^
SFA	1759.88 ± 383.428	1004.18 ± 134.206^*∗*^
Total	3636.6 ± 622.343	1565.47 ± 188.869^*∗*^

Results are based on 8 independent preparations for each experimental treatment (means ± SD). ^*∗*^ *p* < 0.05 compared with control group.

**Table 5 tab5:** Free fatty acids composition in the submandibular salivary glands (nmol/g).

Fatty acid	Control	High fat diet
Myristic (14:0)	50.36 ± 7.533	13.05 ± 2.304^*∗*^
Palmitic (16:0)	1044.21 ± 293.655	136.95 ± 22.83^*∗*^
Palmitoleic (16:1)	136.64 ± 33.283	3.2 ± 0.647^*∗*^
Stearic (18:0)	673.43 ± 215.344	147.14 ± 21.13^*∗*^
Oleic (18:1)	374.01 ± 68.926	21.52 ± 4.774^*∗*^
Linoleic (18:2)	347.78 ± 77.772	11.45 ± 2.765^*∗*^
Arachidic (20:0)	10.32 ± 2.892	5.01 ± 0.88^*∗*^
*α*-linoleic (18:3)	31.6 ± 11.089	1.97 ± 0.495^*∗*^
Behenic (22:0)	11.71 ± 0.945	2.39 ± 0.336^*∗*^
Arachidonic (20:4)	828.63 ± 332.348	5.27 ± 1.259^*∗*^
Lignoceric (24:0)	3.67 ± 0.97	2.53 ± 0.444^*∗*^
Eicosapentaenoic (20:5)	24.19 ± 6.655	1.55 ± 0.656^*∗*^
Nervonic (24:1)	3.78 ± 0.859	1.32 ± 0.417^*∗*^
Docosahexaenoic (22:6)	46.98 ± 7.058	1.1 ± 0.145^*∗*^
UFA	1793.6 ± 488.952	47.38 ± 7.813^*∗*^
SFA	1793.71 ± 515.048	307.07 ± 44.118^*∗*^
Total	3587.31 ± 959.758	354.44 ± 46.163^*∗*^

Results are based on 8 independent preparations for each experimental treatment (means ± SD). ^*∗*^ *p* < 0.05 compared with control group.

**Table 6 tab6:** Diacylglycerols composition in the parotid salivary glands (nmol/g).

Fatty acid	Control	High fat diet
Myristic (14:0)	87.79 ± 22.188	56.03 ± 7.414^*∗*^
Palmitic (16:0)	984.94 ± 210.904	575.82 ± 94.842^*∗*^
Palmitoleic (16:1)	184.17 ± 24.947	49.8 ± 9.994^*∗*^
Stearic (18:0)	431.31 ± 101.976	200.57 ± 29.904^*∗*^
Oleic (18:1)	134.86 ± 21.235	93.98 ± 12.105^*∗*^
Linoleic (18:2)	171.37 ± 35.577	130.41 ± 16.157^*∗*^
Arachidic (20:0)	5.98 ± 1.84	5.05 ± 0.855
*α*-linoleic (18:3)	7.31 ± 1.464	5.26 ± 0.914^*∗*^
Behenic (22:0)	12.08 ± 3.819	3.79 ± 0.715^*∗*^
Arachidonic (20:4)	184.27 ± 76.864	94.84 ± 19.375^*∗*^
Lignoceric (24:0)	3.77 ± 1.47	3.24 ± 0.514
Eicosapentaenoic (20:5)	7.5 ± 3.514	5.38 ± 0.913
Nervonic (24:1)	1.26 ± 0.382	1.31 ± 0.221
Docosahexaenoic (22:6)	28.71 ± 5.259	5.89 ± 0.927^*∗*^
UFA	719.47 ± 127.861	386.87 ± 51.931^*∗*^
SFA	1525.87 ± 303.224	844.5 ± 99.547^*∗*^
Total	2245.33 ± 426.18	1231.37 ± 128.942^*∗*^

Results are based on 8 independent preparations for each experimental treatment (means ± SD). ^*∗*^ *p* < 0.05 compared with control group.

**Table 7 tab7:** Diacylglycerols composition in the submandibular salivary glands (nmol/g).

Fatty acid	Control	High fat diet
Myristic (14:0)	48.38 ± 11.363	38.27 ± 7.62^*∗*^
Palmitic (16:0)	599.62 ± 94.795	281.06 ± 47.48^*∗*^
Palmitoleic (16:1)	58.54 ± 19.612	4.72 ± 0.704^*∗*^
Stearic (18:0)	364.07 ± 54.254	261.05 ± 48.843^*∗*^
Oleic (18:1)	142.47 ± 40.036	44.75 ± 5.25^*∗*^
Linoleic (18:2)	186.84 ± 50.092	43.5 ± 5.515^*∗*^
Arachidic (20:0)	9.04 ± 1.714	6.11 ± 0.856^*∗*^
*α*-linoleic (18:3)	8.62 ± 1.633	3.13 ± 0.448^*∗*^
Behenic (22:0)	7.08 ± 0.724	2.94 ± 0.475^*∗*^
Arachidonic (20:4)	184.5 ± 48.12	35.4 ± 3.616^*∗*^
Lignoceric (24:0)	2.3 ± 0.579	2.14 ± 0.393
Eicosapentaenoic (20:5)	4.65 ± 1.736	2.32 ± 0.334^*∗*^
Nervonic (24:1)	0.75 ± 0.188	1.04 ± 0.211^*∗*^
Docosahexaenoic (22:6)	13.61 ± 2.375	2.06 ± 0.324^*∗*^
UFA	600 ± 134.423	136.92 ± 10.521^*∗*^
SFA	1030.49 ± 155.56	591.57 ± 101.192^*∗*^
Total	1630.49 ± 266.998	728.49 ± 107.393^*∗*^

Results are based on 8 independent preparations for each experimental treatment (means ± SD). ^*∗*^ *p* < 0.05 compared with control group.

**Table 8 tab8:** Triacylglycerols composition in the parotid salivary glands (nmol/g).

Fatty acid	Control	High fat diet
Myristic (14:0)	95.58 ± 16.557	93.13 ± 10.494
Palmitic (16:0)	3753.26 ± 577.212	4106.75 ± 355.387
Palmitoleic (16:1)	725.82 ± 311.454	457.56 ± 60.399
Stearic (18:0)	1545.87 ± 203.585	1739.3 ± 179.592
Oleic (18:1)	740.19 ± 141.33	758.58 ± 60.541
Linoleic (18:2)	1745.84 ± 508.939	2123 ± 239.624^*∗*^
Arachidic (20:0)	6.46 ± 0.973	6.98 ± 1.074
*α*-linoleic (18:3)	15.69 ± 5.458	14.51 ± 3.86
Behenic (22:0)	57.18 ± 12.029	25.77 ± 5.108^*∗*^
Arachidonic (20:4)	2336.43 ± 247.191	2462.9 ± 323.202
Lignoceric (24:0)	6.2 ± 1.302	7.04 ± 1.189
Eicosapentaenoic (20:5)	63.45 ± 20.07	114.81 ± 19.906^*∗*^
Nervonic (24:1)	6 ± 3.685	4.11 ± 2.077
Docosahexaenoic (22:6)	175.64 ± 37.506	250.05 ± 49.041^*∗*^
UFA	5809.07 ± 895.885	6185.53 ± 508.847
SFA	5464.55 ± 774.298	5978.97 ± 530.721
Total	11273.62 ± 1662.308	12164.5 ± 1031.873

Results are based on 8 independent preparations for each experimental treatment (means ± SD). ^*∗*^ *p* < 0.05 compared with control group.

**Table 9:** Triacylglycerols composition in the submandibular salivary glands (nmol/g).

Fatty acid	Control	High fat diet
Myristic (14:0)	72.45 ± 10.702	47.77 ± 3.466^*∗*^
Palmitic (16:0)	2978.17 ± 381.425	2979.54 ± 157.422
Palmitoleic (16:1)	183.65 ± 28.609	67.47 ± 4.61^*∗*^
Stearic (18:0)	1575.82 ± 255.862	2004.72 ± 79.831^*∗*^
Oleic (18:1)	732.15 ± 62.753	953.72 ± 35.941^*∗*^
Linoleic (18:2)	1416.05 ± 347.755	2068.17 ± 272.292
Arachidic (20:0)	14.95 ± 4.395	19.21 ± 3.536
*α*-linoleic (18:3)	15.17 ± 5.569	14.62 ± 1.299
Behenic (22:0)	31.32 ± 9.904	28.75 ± 5.13
Arachidonic (20:4)	2542.74 ± 561.505	2542.69 ± 176.752
Lignoceric (24:0)	15.01 ± 1.78	12.17 ± 1.746^*∗*^
Eicosapentaenoic (20:5)	40.37 ± 13.144	95.49 ± 20.396^*∗*^
Nervonic (24:1)	9.62 ± 4.199	6.56 ± 1.041^*∗*^
Docosahexaenoic (22:6)	171.85 ± 52.848	239.27 ± 29.357^*∗*^
UFA	5111.61 ± 933.047	5988 ± 227.362
SFA	4687.72 ± 623.025	5092.15 ± 206.762
Total	9799.33 ± 1548.921	11080.15 ± 422.14^*∗*^

Results are based on 8 independent preparations for each experimental treatment (means ± SD). ^*∗*^ *p* < 0.05 compared with control group.
